# Changes

**Published:** 1882-06

**Authors:** 


### CHANGES.

Some changes have recently been made in the Faculty of the Baltimore College of Dental Surgery. Prof. F. J. S. Gorgas, who has for twenty-five years occupied a chair in that Institution and for the most of that time been the Dean of the Faculty, has resigned to occupy similar positions in the new Dental Department of the University of Maryland, than which there are but two older medical colleges in this country.

Prof. Jas. H. Harris also resigned his position in the Baltimore Dental College and has accepted a position in the new department. Thus a new Dental Institution comes into existence, and in connection with a University and a Medical College, and much may be expected from it, having for its principal special teachers, two men of long and successful experience.

It is to be hoped that the vacancies thus made will be filled by teachers capable of sustaining the reputation of the Baltimore Dental College. Prof. R. B. Winder has been elected Dean of the Faculty; Dr. M. W. Foster, Professor of Pathology and Therapeutics, and Dr. J. E. Lindsay, Professor of Chemistry.

At a meeting of the Chicago Dental Society, held May 2d, the following preamble and resolutions were unanimously adopted:

*Whereas*, It has pleased the Supreme Ruler of the Universe to call from among us our esteemed friend and associate, the late Dr. D. C. Hawxhurst, of Battle Creek, Mich., whose untimely decease was due to his untiring efforts in the pursuit of professional knowledge, and

*Whereas*, We have been deprived of the companionship and counsel of our brother, whose ability and learning, unsullied reputation and high sense of honor have endeared him to the memory of his associates, it is right and proper that we should show, with what feelings of deep regret and sorrow, we learned of his untimely death, therefore,

*Resolved*, That in the decease of Dr. D. C. Hawxhurst, our profession in general and the Michigan State Dental and Medical Associations in particular, have lost a useful, wise and good brother.

*Resolved*, That his generous, manly character and studious habits commend our admiration, and his example. our earnest following

*Resolved*, That to the family of the deceased we tender our heartfelt sympathy in this, their time of bereavement and mourning.

*Resolved*, That a copy of these resolutions be sent to the family of the deceased, to the Michigan State Dental and Medical Associations, and a copy transmitted for publication to each of the Dental Journals.

T. W. BROPHY,

A. W. HARLAN, } Committee.

GEO. H. CUSHING.

